# Benchmarking multimodal large language models for medicinal plant identification

**DOI:** 10.3389/fpls.2026.1765281

**Published:** 2026-06-26

**Authors:** Yue Jiang, Zhenzhong Dai, Wen Jin, Weinan Lin, Yijun Xu, Jiangda Wang, Zhaoxi Fang

**Affiliations:** 1 Department of Computer Science and Engineering, Shaoxing University, Shaoxing, China; 2 Institute of Artificial Intelligence, Shaoxing University, Shaoxing, China; 3 School of Computing, College of Science, Engineering and Technology, The University of South Africa, Roodepoort, South Africa

**Keywords:** identification accuracy, image recognition accuracy, large language models, medicinal plant, model evaluation

## Abstract

With the rapid advancement of artificial intelligence technology, large language models (LLMs) have shown considerable potential in the medical field. This study systematically evaluated the performance of five multimodal LLMs, including GPT-4o, Llama4Scout, Gemma3-27B, Qwen3-VL235B, and DeepSeek-VL2, in medicinal plant image recognition tasks. We selected 200 medicinal plant images from a privately copyrighted dataset and tested the models using a four-choice format to evaluate their recognition accuracy. Results indicate that Qwen3-VL-235B demonstrated the highest accuracy at 90.50%, outperforming other models. GPT-4o ranked second at 85.00%, while Gemma3-27B, Llama4Scout, and DeepSeek-VL2 achieved 65.50%, 58.50%, and 58.00%, respectively. These findings indicate that multimodal LLMs hold promising potential for medicinal plant recognition, yet further optimization for specific domains is required to enhance accuracy.

## 1 Introduction

In recent years, large language models (LLMs) have made significant progress in natural language processing. From early statistical language models to neural language models based on deep learning, and now to ultra-large-scale pre-trained models, the pace of technological iteration has accelerated rapidly (Li et al., 2024; Zhao et al., 2024; Qin et al., 2024). Pre-trained language models such as the GPT series, BERT, and T5 have acquired powerful language understanding and generation capabilities through unsupervised learning on massive text corpora. Particularly since the successful release of ChatGPT, LLMs have demonstrated unprecedented conversational interaction and knowledge reasoning abilities, sparking a global AI technology revolution (Floridi and Chiriatti, 2020; Zhao et al., 2024).

The application of LLMs in the medical field has become an important direction for the development of AI in healthcare (Bedi et al., 2024; Zhou et al., 2025). Medicine, as a knowledge-intensive professional field, contains vast amounts of professional text data such as medical literature, clinical guidelines, and case reports, providing a rich data foundation for LLM applications. Currently, LLM applications in medicine mainly include medical question answering, clinical decision support, medical document processing, medical record analysis, drug development, and drug interaction analysis (Dai et al., 2024; Zhou et al., 2025). LLMs can learn from extensive medical literature and clinical guidelines to provide evidence-based diagnosis and treatment recommendations for doctors. Research shows that advanced medical-specific LLMs perform close to or even exceed the average level of human doctors in medical examinations and disease diagnosis, demonstrating strong medical knowledge mastery (Bedi et al., 2024; Zhou et al., 2025). LLMs can automatically extract key information from medical records, generate structured medical data, and assist doctors in writing and reviewing medical records. Meanwhile, models can also discover disease patterns and treatment rules from large volumes of medical records, supporting clinical research and quality improvement. Furthermore, LLMs play an important role in medical education and training. Models can generate personalized learning materials, simulate doctor-patient dialogue scenarios, and provide standardized training and assessment tools for medical students and residents (Zhou et al., 2025).

However, traditional LLMs are primarily limited to text modality and face clear limitations when handling multimodal information involving vision, audio, etc. Multimodal large language models (MLLMs) combine visual encoders with language models to enable simultaneous understanding and processing of text, images, videos, and other modalities, significantly expanding the application scope of large language models, especially in medical image analysis (Dai et al., 2024; Zhou et al., 2024; Lin et al., 2025; Tian et al., 2024; Zhou et al., 2021). MLLMs can simultaneously process medical images and related text information, enabling more intelligent and accurate medical image analysis (Bluethgen et al., 2025; Ji et al., 2024; Chen et al., 2024). For example, LLMs can analyze chest X-rays, CT scans, and other images, identify abnormalities such as lung nodules, fractures, and tumors, and generate structured diagnostic reports (Bluethgen et al., 2025; Thawkar et al., 2023; Yang et al., 2023). Some studies indicate that MLLMs have achieved performance comparable to or exceeding that of human radiologists in specific radiology tasks (Miao et al., 2024; Yang et al., 2023).

While previous studies have explored LLMs in medical imaging, few have systematically evaluated their performance in fine-grained botanical tasks such as medicinal plant identification. Medicinal plant identification is an important research area at the intersection of medicine and botany, with profound academic value and practical significance. Traditionally, medicinal plant identification relies on expert experience and visual observation, which suffers from issues such as strong subjectivity, low efficiency, and lack of standardization. With the development of AI technology, using computer vision for automatic medicinal plant identification has become a research hotspot (Bisen, 2021; Oppong, 2022; Wang et al., 2022; Sachar et al., 2022; Sun et al., 2022).

Evaluating the medicinal plant identification capability of LLMs has important theoretical and practical significance. As a typical fine-grained visual classification task, it highly demands visual understanding and knowledge reasoning abilities, providing a testbed for examining MLLM practical capabilities (Luo et al., 2024). Accurate medicinal plant identification is crucial for traditional Chinese medicine (TCM) quality control, drug development, and ecological protection (Azadnia and Kheiralipour, 2021; Fouziya et al., 2025). Automated medicinal plant identification systems can greatly improve the efficiency and accuracy of Chinese herbal medicine authentication, reduce human errors, and ensure medication safety (Wang et al., 2022; Sun et al., 2022).

This study systematically evaluates the performance of several representative LLMs in medicinal plant identification tasks, aiming to provide a scientific basis and practical guidance for the development of related technologies and promote the deep application of AI in the field of TCM (Liu et al., 2023; Wang et al., 2022; Sun et al., 2022). This study selects five popular multimodal LLMs: GPT-4o (Miao et al., 2024), Llama4Scout (Meta AI, 2025), Gemma3-27B (Google DeepMind, 2025; Google, 2024), Qwen3-VL-235B (Yang et al., 2025), and DeepSeek-VL2 (Wu et al., 2024) for evaluation. We select 200 medicinal plant images from a privately copyrighted dataset as test samples and use single-choice questions to test the five models. Each test sample contains four options to evaluate the recognition accuracy. The test results show significant differences in performance among the models. Qwen3-VL-235B performs the best, achieving an accuracy of 90.50%; GPT-4o ranks second with an accuracy of 85.00%; while Gemma3-27B, Llama4Scout, and DeepSeek-VL2 have lower accuracies. Overall, multimodal LLMs show good potential in medicinal plant identification tasks, but performance varies significantly across models.

## 2 Methods

### 2.1 Medicinal plant image dataset

The medicinal plant image dataset used in this study is a privately copyrighted resource (CNBLOG, 2025), which contains 880 medicinal plant images covering various common medicinal plants, including traditional Chinese medicinal herbs and folk medicinal plants, possessing strong representativeness and practical value. The dataset includes a rich variety of medicinal plant species, covering plants from different families and genera, including herbs, woody plants, vines, and other growth forms. These plants exhibit certain differences in morphological characteristics, color, texture, etc., providing a good test basis for evaluating model recognition capabilities.

In this study, 200 images were selected as the test set, covering 68 common medicinal plant species. Stratified sampling was adopted to ensure species diversity. We manually inspected and removed duplicate and near-duplicate images to eliminate data redundancy and potential evaluation bias. The selected test images cover multiple morphological types, including plant leaves, flowers, roots, and stems. All category labels were carefully verified by experts to ensure taxonomic accuracy. The number of sampled images per species is relatively balanced.

### 2.2 Tested LLMs

This study selects five representative multimodal LLMs for testing, as shown in **Table 1**. These models have distinct characteristics in terms of architecture design, parameter scale, training methods, etc., comprehensively reflecting the current development level of multimodal LLM technology.

**Table 1:** LLMs tested in this study.

Model	Architecture type	Total params	Context length
GPT-4o	Dense	Not disclosed	128K
Llama4Scout	MoE	109B	10M
Gemma3-27B	Dense	27B	128K
Qwen3-VL-235B	MoE	235B	32K
DeepSeek-VL2	MoE	Not disclosed	32K

#### 2.2.1 GPT-4o

GPT-4o is the latest generation multimodal LLM developed by OpenAI, representing the top level of current LLM technology (Yang et al., 2023). This model has undergone significant improvements based on GPT-4, with stronger multimodal understanding capabilities and higher processing efficiency (Yang et al., 2023). In the field of medical image analysis, GPT-4o has demonstrated excellent performance, accurately identifying abnormal signs in medical images and generating professional diagnostic reports (Miao et al., 2024; Yang et al., 2023).

#### 2.2.2 Llama4Scout

Llama4Scout is a lightweight version in the Llama 4 series developed by Meta, specifically designed for efficient inference and deployment optimization (Meta AI, 2025). This model adopts an innovative mixtureof-experts (MoE) architecture, significantly reducing computational costs while ensuring performance. Llama4Scout performs excellently in multiple benchmark tests, showing particular advantages in multidocument summarization and long-text understanding tasks.

#### 2.2.3 Gemma3-27B

Gemma3-27B is the latest version of the Gemma series developed by Google DeepMind, a lightweight yet powerful multimodal LLM (Google DeepMind, 2025). Gemma3-27B integrates a 400M parameter SigLIP visual encoder, supporting image processing at 896×896 resolution. Gemma3-27B performs excellently in visual language tasks, particularly demonstrating strong capabilities in document understanding and chart analysis tasks. Its balanced performance and efficiency make it an ideal test subject for medicinal plant identification tasks.

#### 2.2.4 Qwen3-VL-235B

Qwen3-VL-235B is a multimodal LLM from the Qwen series developed by Alibaba DAMO Academy, specifically optimized for visual language tasks (Yang et al., 2025). This model has unique advantages in Chinese understanding and multimodal fusion. Qwen3-VL-235B has a total parameter count of 235B, with 22B activated parameters, possessing high representational capacity. Qwen3-VL-235B performs excellently in Chinese visual language tasks, particularly suitable for handling multimodal tasks involving Chinese professional terminology and knowledge.

#### 2.2.5 DeepSeek-VL2

DeepSeek-VL2 is a multimodal LLM developed by DeepSeek, adopting a mixture-of-experts architecture and demonstrating excellent performance in visual understanding tasks (Wu et al., 2024). DeepSeekVL2 adopts a sparsely activated mixture-of-experts architecture to improve computational efficiency and supports dynamic tiling visual encoding strategies to adapt to images of different resolutions. DeepSeekVL2 performs excellently in multiple visual language benchmark tests, showing strong competitiveness especially in Chinese visual understanding tasks.

Among these models, GPT-4o is a closed-source dense model with strong general multimodal capabilities but undisclosed parameter scale. Llama4Scout, Qwen3-VL-235B, and DeepSeek-VL2 adopt the MoE architecture to balance performance and efficiency, while Gemma3-27B uses a lightweight dense structure for better deployment compatibility. In terms of language and domain adaptation, GPT-4o and Gemma3-27B are primarily English-centric general models, whereas Qwen3-VL-235B and DeepSeek-VL2 are optimized for Chinese scenarios, making them more suitable for medicinal plant identification involving traditional Chinese medicine terminology.

### 2.3 Experimental settings and reproducibility

All five multimodal models in this study were tested using their official public APIs, and the official default parameter settings were used to ensure experimental reproducibility, including temperature, top-p, maximum output token count, and random seed. All evaluations adopted the official stable version of each model with a unified access period in October 2025, and inference was implemented via the officially authorized standard API endpoint of each platform. The identical prompt template was used for all models throughout the test, and all medicinal plant names in prompts and candidate options were presented only in standard Chinese common names without Latin or English nomenclature, which is consistent with the practical scenario of Chinese herbal plant identification. In each four-choice question, one correct medicinal plant species was matched with three distractor species randomly selected from the whole dataset. All distractors were real medicinal plant species, maintaining a consistent and fair evaluation setting across all test samples.

During the whole evaluation, each image was tested only once to avoid repeated sampling bias, and all models were required to output merely the option label of A/B/C/D without additional reasoning or descriptive explanations. Any ambiguous, off-topic, or non-compliant outputs that did not follow the specified answer format were uniformly judged as incorrect recognition according to a standardized rule.

## 3 Results

**Figure 1:**
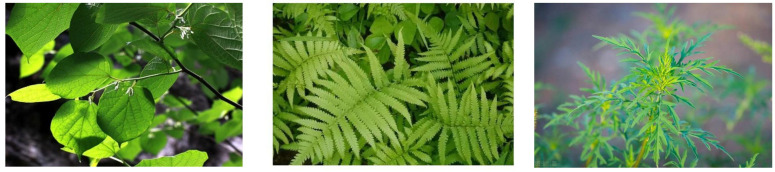
Several medicinal plant test images.

**Figure 2:**
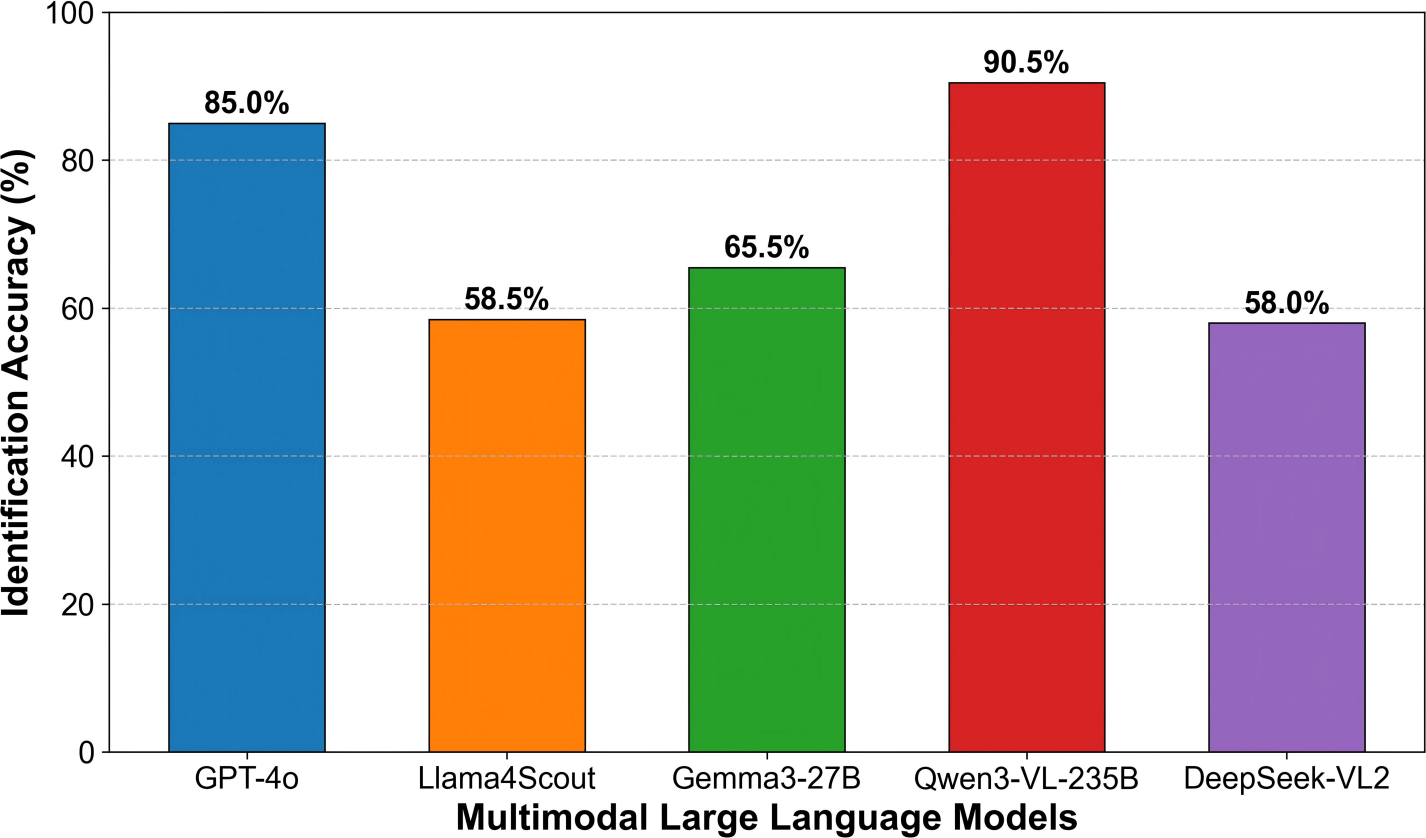
Medicinal plant identification results.

We select 200 plant images from the dataset for testing. **Figure 1** shows several typical medicinal plant test images. Through the identification test of 200 medicinal plant images, we obtain the overall performance of the five LLMs. The results are shown in **Figure 2**.

From the results, it can be seen that Qwen3-VL-235B performs the best, with an accuracy rate of 90.50%, outperforming other evaluated models. One possible reason for this result is that the model was trained on a large amount of Chinese medical data, giving it a natural advantage in understanding knowledge related to medicinal plants. GPT-4o ranks second with an accuracy rate of 85.00%, reflecting its powerful capability as a general multimodal large model. Although not specifically optimized for medicinal plant identification, its excellent visual understanding ability and broad knowledge base still allow it to perform well in this task. Gemma3-27B has an accuracy rate of 65.50%, at a medium level. As a relatively lightweight model, this performance is quite remarkable, showing Google’s technical strength in model efficiency optimization. Llama4Scout and DeepSeek-VL2 have accuracy rates of 58.50% and 58.00%, respectively, performing relatively poorly. This may be related to the limitations of these models in visual understanding ability, especially when dealing with fine-grained plant classification tasks.

**Figure 3:**
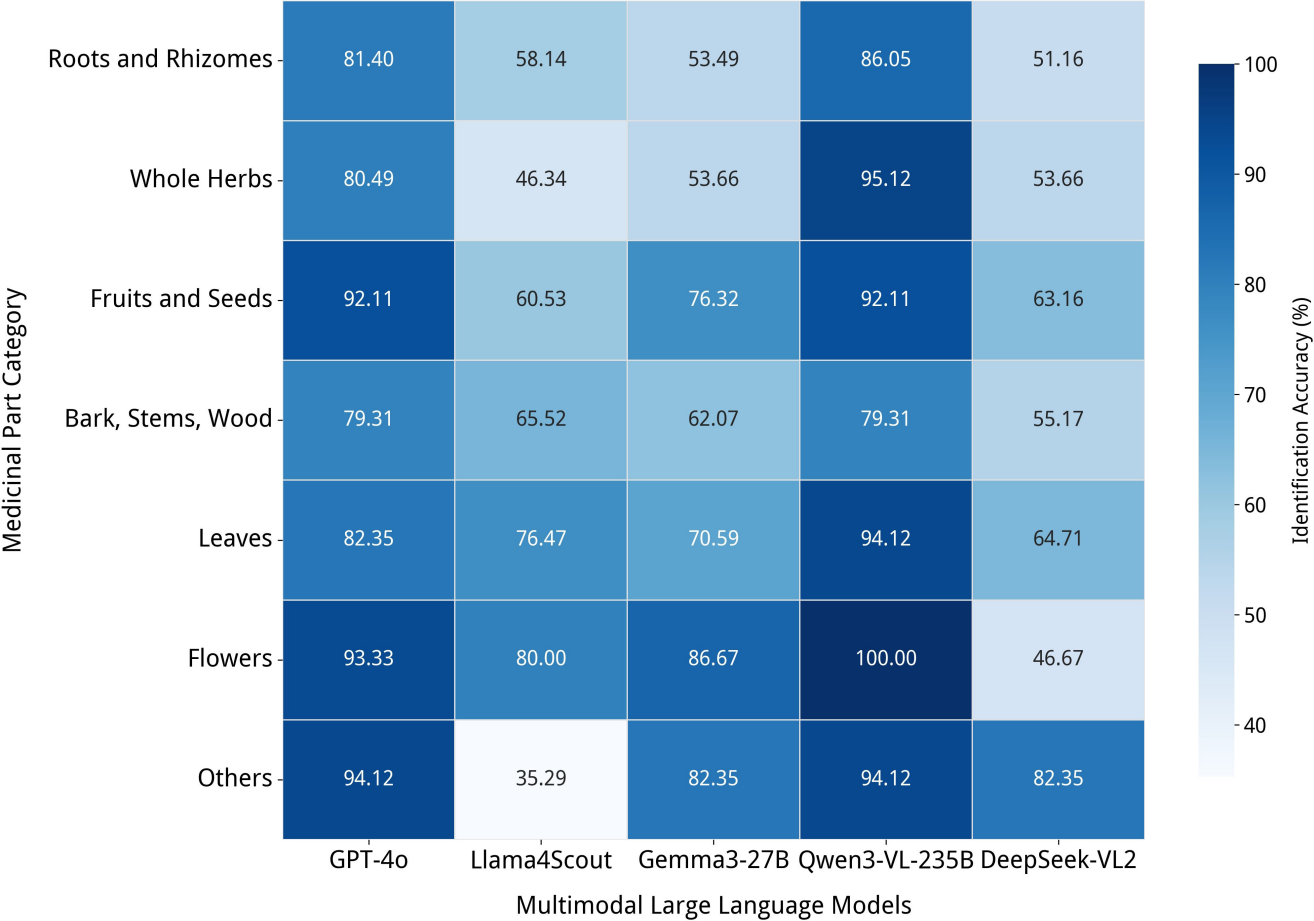
Identification accuracy of MLLMs across medicinal plant categories.

To comprehensively compare model performance on different medicinal plant types, all 200 test images were divided into seven categories according to medicinal parts, and the identification accuracy of each model was visualized via a heatmap (**Figure 3**). Across all categories, flower samples obtained the highest recognition accuracy, where Qwen3-VL-235B reached 100.00% due to unique and distinguishable floral morphological features. By contrast, root and rhizome samples presented the lowest average accuracy across models, as their high interspecies morphological similarity increases fine-grained identification difficulty. Fruit–seed and leaf categories maintained generally stable and high accuracy; Qwen3-VL-235B showed a prominent advantage in whole herb identification, whereas bark, stem and other types achieved moderate and relatively balanced performance among all evaluated models.

By analyzing the identification results of different medicinal plants, we found that some plants were relatively easy to identify, while others presented greater identification difficulties. **Table 2** shows the top 10 medicinal plants with the highest recognition error rates. Further botanical analysis was conducted to explore the causes of recognition errors. The top three species with 100% error rates, namely Anoectochilus roxburghii, Salvia przewalskii, and Berberis spp., exhibit extremely similar morphological characteristics with their congeneric and familial relatives, showing only subtle differences in leaf shape, vein texture and overall appearance. Another seven species including Lemna minor, Sophora tonkinensis, Dictamnus dasycarpus, Dipsacus asperoides, Anisomeles indica, Rhodomyrtus tomentosa, and Sauropus spatulifolius also reach an error rate of 80%. Their low distinguishability mainly originates from inconspicuous local features and high morphological similarity with adjacent taxa.

**Table 2:** Top 10 medicinal plants with highest recognition error rates.

Rank	Plant name (Latin/scientific)	Error rate
1	Anoectochilus roxburghii	100.0%
2	Salvia przewalskii	100.0%
3	Berberis spp.	100.0%
4	Lemna minor	80.0%
5	Sophora tonkinensis	80.0%
6	Dictamnus dasycarpus	80.0%
7	Dipsacus asperoides	80.0%
8	Anisomeles indica	80.0%
9	Rhodomyrtus tomentosa	80.0%
10	Sauropus spatulifolius	80.0%

Besides taxonomic similarity, recognition accuracy is also affected by multiple factors, such as different plant organs shown in images, varying shooting angles, image quality, and complex phenological stages. In contrast, a total of 36 medicinal plant species represented by Auricularia auricula-judae, Hibiscus syriacus, and Artemisia annua achieve 100% recognition accuracy across all models, due to their unique and highly distinguishable morphological traits that are not easily confused with other species.

## 4 Discussion

The performance variations observed among models in this test reflect distinct characteristics in current multimodal LLMs regarding architectural design, training strategies, and adaptability to application scenarios. The architectural designs adopted by different models impact their performance in medicinal plant identification tasks. High-performing models like Qwen3-VL-235B and GPT-4o typically employ more advanced vision-language fusion architectures, enabling them to integrate visual features with linguistic knowledge more effectively. These models prioritize deep multimodal information fusion in their design, rather than simple modal concatenation. In contrast, models with relatively weaker performance may exhibit deficiencies in visual encoder design or incomplete multimodal fusion mechanisms.

One possible explanation for the superior performance of Qwen3-VL-235B is its better adaptation to Chinese medicinal and botanical knowledge accumulated during pre-training. Other potential influencing factors include model parameter scale, inherent capability of visual encoder, and different sensitivity to Chinese and Latin plant nomenclature. Further controlled ablation experiments are required in future work to explore the internal mechanism behind the performance difference.

Compared with traditional computer vision models that rely only on supervised training over fixed image datasets, multimodal large language models possess inherent open-set knowledge and cross-modal reasoning capability. Conventional classification models such as ResNet, EfficientNet, ViT and Swin Transformer mainly depend on large-scale labeled medicinal plant images for fine-tuning, and their generalization ability is limited by dataset scale and label quality. In contrast, multimodal LLMs leverage broad pre-trained botanical and herbal knowledge, showing distinct advantages in dealing with visually similar and taxonomically close medicinal plant species.

In the context of Sustainable and Intelligent Phytoprotection, accurate medicinal plant identification is of great practical significance for biodiversity conservation and sustainable utilization of botanical resources. Reliable species recognition provides basic technical support for the protection of endangered medicinal plants and the rational conservation of wild plant resources. Misidentification of morphologically similar species may easily cause herbal adulteration, affect clinical medication safety, and bring hidden dangers to the standard circulation of medicinal plant supply chains. The multimodal identification method explored in this study has good application potential in wild resource field surveys, germplasm collection and documentation, as well as daily supervision of Chinese herbal medicine circulation.

This study has certain limitations. This study mainly focuses on the horizontal performance comparison of mainstream multimodal LLMs under a unified four-choice identification paradigm. The current task setting is relatively simplified compared with real open-set medicinal plant identification. In addition, this manuscript does not include statistical significance verification, confidence interval analysis, and quantitative comparison with traditional computer vision baselines. These extended evaluations will be supplemented and explored in our future in-depth research.

## 5 Conclusion

This study evaluates five popular multimodal LLMs on the task of medicinal plant identification, providing crucial empirical data and a reference for theoretical research in related fields. The test results show significant differences in the performance of different models in medicinal plant identification tasks. Qwen3-VL-235B ranked first with an accuracy of 90.50%, GPT-4o ranked second with an accuracy of 85.00%, while Gemma3-27B, Llama4Scout, and DeepSeek-VL2 had relatively lower accuracies. This result reflects the differences in visual understanding ability and professional knowledge mastery among different models. The excellent performance of Qwen3-VL-235B may be attributed to its better adaptation to Chinese medical and botanical knowledge accumulated during pre-training. This finding indicates that models optimized for specific languages and domains have clear advantages in professional tasks, providing important insights for future model development. With continuous technological progress and expanding application scenarios, we believe this technology will play an increasingly important role in TCM modernization, ecological protection, health education, and other fields, making important contributions to human health and sustainable development.

## Data Availability

The original contributions presented in the study are included in the article/supplementary material. Further inquiries can be directed to the corresponding author.
